# Effect of baker’s yeast fermented moist feed on the growth and bone mineralization in broiler

**DOI:** 10.5455/javar.2024.k750

**Published:** 2024-03-30

**Authors:** Jesmin Aktar, Khan Md. Shaiful Islam, Rakhi Chowdhury, Momota Rani Debi, Ashik Iqbal Emon

**Affiliations:** 1 Department of Animal Nutrition, Bangladesh Agricultural University, Mymensingh, Bangladesh; 2 Department of Poultry Science, Bangladesh Agricultural University, Mymensingh, Bangladesh

**Keywords:** Broiler, fermentation, feed, performance, yeast

## Abstract

**Objective:**

The effect of feeding yeast-fermented feed in various forms on broiler growth performance and bone mineralization was studied.

**Materials and Methods:**

Initially, a corn-soy-based diet was formulated and fermented in anaerobic conditions at 28°C in laboratory space for 48 h with yeast (2.0%) and moisture (50%). Afterward, the 150 newly hatched Arbeor Acres commercial broiler chicks were divided into 5 dietary groups (30 chicks, 6 cages, and 5 birds per cage). Each group received one of the following formulated and fermented diets: dry feed (DF), moist feed (MF), yeast-added dry feed (Y-DF), yeast-added moist feed (Y-MF), or yeast-fermented moist feed (YF-MF). Water and feed were supplied *ad libitum.* Six birds per group were slaughtered at age 37 for the determination of carcass traits and tibia ash.

**Results:**

Fermentation improved crude protein from 20.7% to 22.8% but declined crude fiber from 7.9% to 6.3% in the YF-MF group compared to the DF group. High body weight gain was recorded in 771, 830, and 992 gm in the MF, Y-MF, and YF-MF groups, respectively, compared to the DF (762 gm) group (*p* < 0.01). The feed conversion ratio was better in the Y-MF (1.57) and YF-MF (1.57) groups than in the DF (1.75) group. Feeding a fermented, moist diet resulted in improved carcass yield (69%) in the YF-MF group. Bone mineralization expressed a better tibia ash percentage (35% from 30%) in the YF-MF group compared to the DF group.

**Conclusion:**

Therefore, YF-MF enhanced the quality of feed and improved growth, carcass weight, and bone mineralization in broiler.

## Introduction

The poultry industry contributes two major food products (eggs and meat) to fulfill the protein requirement of human alimentation. Because of this, the efficacy of chicken production in converting feed into meat is crucial. But feed contributes a major part of the total production cost in commercial broiler production. It is therefore imperative that the quality of feed ingredients be improved through the use of various techniques, like the fermentation of feed, which is gaining popularity [1]. Fermentation is mostly used to convert sugars and other carbohydrates into useful end products, which also increase the number of microbial cells in the substrate [2].

Fermentation using any type of microbes increases the number of a single cell which acts as a probiotic and modifies the gastrointestinal microbial community to favor the nutritive value of feed and reduce susceptibility against disease [3]. Due to fermentation, ethanol is an end product that contains higher energy than the original composition [4]. Fermentation also improves crude protein content and quality of protein in fermented material which is related to higher biological value through increased utilization of protein in the body [5]. It improves the sensory quality of the feed and makes it safe for animals by degrading toxic components and anti-nutritive factors, producing antioxidants and antibacterial substances [6]. To improve poultry production, yeast as probiotics have been demonstrated to be more effective than other probiotics for fermentation [7].

Baker’s yeast (*Saccharomyces cerevisiae)* is extensively used in animal nutrition to enhance the growth performance of broilers [8]. Several researchers found that the cell wall of yeast is rich in ß-glucan and mannan oligosaccharides (MOS) that have a positive effect on the development of intestinal villi that can regulate the immunity of the host [9]. Fermentation with baker’s yeast decreases phytate and other anti-nutritional elements in the feed while also increasing nutrient availability for the animal [10]. For the fermentation of the feed, only the yeast is not enough, but the moisture content is also important to manage, which is between 50% and 70% [11]. However, it is questionable whether drying fermented feed is economical or not, as it is time-consuming and adds some extra cost for removing moisture. If it is not economical, then moist fermented feed may be a useful option to offer the birds.

Earlier studies have reported that feeding *Bacillus licheniformis* fermented moist feed improves feed utilization and performance of birds [12]; where several experiments have found the beneficial effect of fermented moist feed on pigs, ducks, and geese [13]. However, limited information is available on poultry, particularly broilers. Considering these facts current research aims to elucidate the effect of dry, moist, and *S. cerevisiae* fermented moist diet on growth performance and bone mineralization.

## Materials and Methods

### Ethical approval

The Animal Welfare and Experimental Ethics Committee of Bangladesh examined and approved the experimental protocols, animal care, and sample collection. [AWEEC/BAU/2023(47)].

### Feed formulation, fermentation, and chemical analysis

Feed components and yeast (*S. cerevisiae*) were purchased from the nearby market, where yeast was imported from China by Angel Yeast Co. Ltd. Initially, starter diets were formulated as a form of mash using a horizontal mixer. Then, 2.0% yeast and 50.0% moisture were added to the formulated ration and also fermented anaerobically at room temperature (26°C–28°C). After fermentation, moist fermented feed was collected for chemical analysis. Before and after fermentation, pH was observed. The proximate components were analyzed using the following method [14]. There were five dietetic groups: 1. dry feed (DF), 2. moist feed (MF), 3. yeast-added dry feed (Y-DF), 4. yeast-added moist feed (Y-MF), and 5. yeast-fermented moist feed (YF-MF). Yeast was added at a 2.0% level in all the groups except the DF and MF groups, where 50% water was added in the MF, Y-MF, and YF-MF dietary groups, respectively.

### Feeding trials and bird management

The feeding trial was performed on straight-run broiler chicks (*Arbeor Acres*) for 37 days on 150 birds in total. The chicks were randomly divided into 5 dietary groups (30 chicks, 6 cages, and 5 birds per cage). Considering animal welfare, birds were kept in the floor cage, and sawdust was spread on the floor as bedding materials.

Diets for broiler chickens were typically designed with 22.0% protein in the initial feed and 19.0% in the finisher feed. In this study, a single diet was considered throughout the period and contained an average amount of protein and energy (Table 1). Different forms of corn-soy-based single diets were offered throughout the period without considering different phases to avoid the effect of feed as a factor, which is followed by different high-impact journals [10,15]. Vaccinations against infectious bursal disease (10 and 21-day) and New Castle Disease (3 and 18-day) were conducted. Live weight, feed offered, feed refusal, and mortality were recorded weekly. After the end of the trial, randomly selected birds from each replication were slaughtered to evaluate carcass traits and tibia ash.

### Collection of tibias and analysis

At 37 days of age, tibia samples were collected for analysis. According to Brenes et al. [16], the tibia was dried at 105°C for 12 h and burned at 550°C, and the ashes were weighed and calculated as tibia ash.

### Cost-benefit analysis

The economic study of broiler production was based on comparing the cost of the ration with the current market price of the feed ingredients at the time of purchase. The feed cost per kilogram of live weight, the production cost per kilogram of live weight, and the relative cost-benefit ratios of the diets compared to the control diet were calculated.

### Statistical analysis

Raw data were recorded using a computer-based Excel program. Then the statistical program "IBM SPSS Statistics 26" was used to examine all the collected and computed data. A one-way analysis of variance was performed in a completely randomized design to determine the significance of group effects. A comparison of group means was performed using Turkey’s honestly significant difference test. Significance was designed at the level of 5% significance (*p* < 0.05).

## Results

### Chemical composition of feed after fermentation

**Figure 1.**
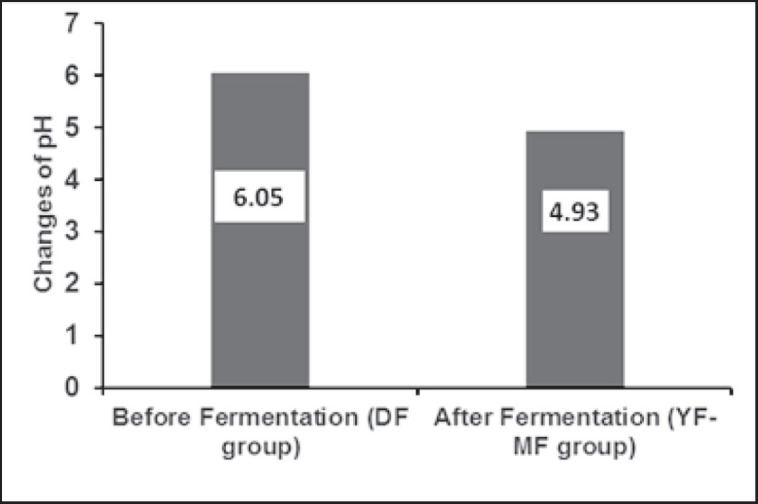
Changes in pH after fermentation.

In the case of feed fermentation, pH is a crucial parameter. In the YF-MF group, fermentation reduces pH from 6.05 to 4.93 compared to the control (DF) group. The result is shown in Figure 1. The chemical composition of feed was affected in all fermented and non-fermented moist groups (Table 2). Protein levels were 21.2%, 21.3%, and 22.8% in the Y-DF, Y-MF, and YF-MF groups, respectively, compared to the DF (20.7%) group. Ether extract increased from 3.0% to 4.5% and ash from 6.9% to 8.9% after fermentation in the YF-MF group compared to the DF group. Due to fermentation, crude fiber decreased from 7.9% to 6.3%, and nitrogen-free extract (NFE) declined from 61.4% to 58.9% in the YF-MF group compared to the DF (control) group.

**Table 1.** Composition and calculated nutritive value of control starter diet.

Ingredient	Amount (%)	*Composition	Amount (%)
Maize	55.56	*ME (kcal/kg)	2,909.99
Soybean meal	32.00	CP%	20.00
Rice bran	9.00	▪Ca%	0.691
DCP	1.00	▪Available P%	0.627
Oil	0.90	Met %	0.520
Limestone	1.14	Lys %	1.075
Salt	0.10		
Methionine	0.20		
Lysine	0.00		
^a^Vit-min premix	0.10		
Total	100		

*Calculated using the formula of (Wiseman, 1987); ▪According to NRC, 1994.^a^2.5 gm Vitamin mineral premix: Vitamin A, 13.500 I.U; Vitamin D3, 1.500 I.U; a-DL-Tocopherolacetate, 50 mg; Menadion, 2 mg; Thiamine, 3 mg; Riboflavin, 5 mg; Pyrodoxin, 4 mg, Cobalamine, 15 µg; Folsaure, 200 µg; Nicotenic acid, 60 µg: Ca-pantothenate, 30 mg; Cholin, 750 mg, Ascorbic acid, 150 mg.

### Growth performance

The starting weight of the birds was 250 gm, but the final body weight was higher in the YF-MF group (1,242 gm) than in the control group (1,012 gm). An increasing trend of weight gain was observed in the MF, Y-MF, and YF-MF groups. Feed intake (FI) was 16.7% higher in group YF-MF than in group DF. The feed conversion ratio (FCR) was best in the Y-MF group and YF-MF group, where broilers were fed a yeast-supplemented moist diet and a yeast-fermented moist diet, respectively. The FCR was a maximum of 10.28% lower in the Y-MF and YF-MF groups than in the control diet (DF).

### Carcass characteristics

Kidney, shank, and liver percentages were increased (*p* < 0.05), but heart percentages were not significant for all dietary groups (Table 4). Feeding a fermented moist diet resulted in a higher dressing yield (69.49%) in comparison to other groups but was not significant.

### Percent tibia ash at 37 days of age

**Figure 2.**
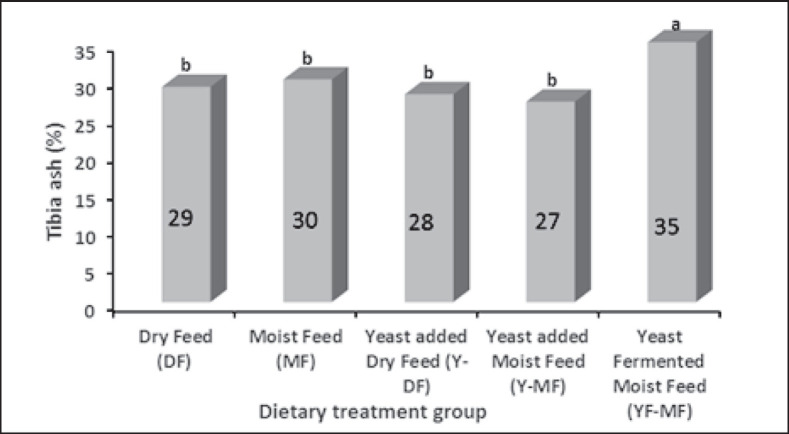
Percent tibia ash at 37 days of age.

Tibia ash percent for all dietary groups ranged from 23% to 35% (Fig. 2). At 37 days of age, tibia ash content was highest (35%) in the YF-MF group (*p* < 0.01), followed by MF (30%), DF (29%), Y-DF (28%), Y-MF (27%), and YF-MF (25%).

### Cost-benefit analysis

The feed cost per kilogram of live body weight was highest in the Y-DF group (0.55$) and was lowest in the MF group (0.43$) (*p* < 0.01) (Table 5). The production cost per kilogram of live body weight was highly significant (*p* < 0.01) for all dietary groups and ranged from 0.96 to 1.08 dollars. The profit margin was highest in the YF-MF dietary group (0.34$) compared to the DF (0.28$) group.

## Discussion

The pH of the fermented feed was reduced, possibly due to the conversion of sugar molecules to an equimolar combination of organic acids, ethanol, and carbon dioxide by the fermentative activity of yeast in the closed medium [17–19]. Fermentation of hand mix feed for 48 h increased CP, EE, and ash, but decreased CF and NFE (Table 2), which supports the findings of Debi et al. [19] and Debi et al. [20]. According to Shuvo et al. [21], the microbial biomass of the fermented product may increase crude protein content, whereas Azrinnahar et al. [10] stated that yeast (*S. cerevisiae*) are single-cell proteins and enhance their activities and multiplies in a proper environment, which may increase the number of peptides and free amino acids in fermented feed [10,22]. The presence of phytate in raw feed ingredients decreases the bioavailability of mineral content. During the fermentation process, yeast can produce a phytase enzyme that degrades phytate and makes the mineral content available [10]. The decline in crude fiber and NFE content might be due to the microorganisms involved in fermentation having the ability to metabolize the fiber components and use soluble sugar as a carbon source that exists in raw grains [23].

**Table 2.** Chemical composition of the diet (gm/100 gm DM).

Parameters (%)	DF	MF	Y-DF	Y-MF	YF-MF
Dry matter	100 ± 0.0	100 ± 0.0	100 ± 0.0	100 ± 0.0	100 ± 0.0
Crude protein	20.7 ± 2.3	20.9 ± 1.9	21.2 ± 2.9	21.3 ± 2.0	22.8 ± 2.7
Crude fibre	7.9 ± 0.8	7.4 ± 0.1	7.6 ± 0.2	6.7 ± 0.5	6.3 ± 0.5
Ether extract	3.0 ± 0.2	1.7 ± 0.3	3.5 ± 0.2	2.2 ± 0.4	4.5 ± 0.2
NFE	61.4 ± 0.6	63.8 ± 4.6	58.9 ± 2.2	60.7 ± 0.0	58.9 ± 0.2
Ash	6.9 ± 0.7	6.6 ± 2.3	8.2 ± 0.7	8.5 ± 1.1	8.9 ± 2.2

**Table 3.** Effect of feeding yeast *S. cerevisiae* fermented moist feed on growth performance and feed efficiency of broiler from day 10–37 (*n* = 30).

Item	Groups					
	**DF**	**MF**	**Y-DF**	**Y-MF**	**YF-MF**	**Sig.**
IBW	250 ± 1.2	250 ± 2.8	250 ± 2.3	250 ± 2.1	250 ± 2.7	NS
FBW	1,012^c^ ± 21.0	1,021^c^ ± 2 4.4	1,011^c^ ± 15.5	1,080^b^ ± 31.2	1,242^a^ ± 29.9	**
BWG	762^c^ ± 20.9	771^c^ ± 24.7	761^c^ ± 17.0	830^b^ ± 30.9	992^a^ ± 28.9	**
FI	1,335^b^ ± 24	1347^b^ ± 13	1317^b^ ± 37	1303^b^ ± 19	1559^a^ ± 32	**
FCR	1.75^a^ ± 0.05	1.75^a^ ± 0.06	1.73^a^ ± 0.08	1.57^b^ ± 0.07	1.57^b^ ± 0.06	**

**= highly significant at 1% level (*p* < 0.01); N.S = Not significant; ^a,b,c,d^Values of different variables under different programme indicates (Mean ± SD). DF: Dry Feed; MF: Moist Feed; Y-DF: Yeast added Dry Feed; Y-MF: Yeast added Moist Feed; YF-MF: Yeast Fermented Moist Feed; IBW: Initial Body weight; FBW: Final Body Weight; BWG: Body Weight Gain; FI: Feed Intake; FCR: Feed Conversion Ratio (kg LWG/ kg FI) SD: Standard Deviation.

**Table 4.** Carcass characteristics (% live weight) of broilers receiving dietary groups for 37 days of age (*n* = 30).

Item	Groups					
	DF	MF	Y-DF	Y-MF	YF-MF	Sig.
Heart (%)	0.51 ± 0.1	0.53 ± 0.1	0.74 ± 0.1	0.74 ± 0.1	0.61 ± 0.12	NS
Liver (%)	1.83^b^ ± 0.4	2.31^ab^ ± 0.4	1.99^ab^ ± 0.3	2.12^ab^ ± 0.3	2.46^a^ ± 0.3	*
Kidney (%)	1.37^b^ ± 0.4	1.38^b^ ± 0.3	1.69^ab^ ± 0.3	1.86^a^ ± 0.3	1.65^ab^ ± 0.2	*
Shank (%)	3.40^b^ ± 0.7	3.84^ab^ ± 0.4	4.08^ab^ ± 0.3	4.36^a^ ± 0.5	3.94^ab^ ± 0.4	*
Dressing yield (%)	67.23 ± 4.7	67.79 ± 3.0	66.01 ± 5.1	67.77 ± 1.3	69.49 ± 14.8	NS

*= significant at 5% level (*p* < 0.05); **= highly significant at 1% level (*p* < 0.01); N.S =Not significant; ^a,b^Means bearing uncommon superscripts in a row differ significantly. DF: Dry Feed; MF: Moist Feed; Y-DF: Yeast added Dry Feed; Y-MF: Yeast added Moist Feed; YF-MF: Yeast Fermented Moist Feed; LBW: Live Body Weight; DY: Dressing Yield; Values of different variables under different program indicates (Mean ± SD).

As expected, YF-MF increased growth performance compared to DF (Table 3) because *S. cerevisiae* supplementation has an impact on the digestive tract, increasing digestive enzymes (protease, amylase, and lipase), which might improve the digestion and absorption rate of dietary nutrients and ultimately increase live weight gain [24]. During the fermentation of feed, yeast secretes various enzymes that make the nutrients available for broiler, and other secretions from the yeast cell wall, like manna oligosaccharides and fructo-oligosaccharides, suppress the action of pathogenic organisms [10]. According to Zhang et al. [25], *S. cerevisiae* acts as a biodegrading agent that increases the length of villi, reduces intestinal pH, reduces intestinal bacteria, boosts the secretion of auxiliary digestive enzymes, and improves nutritional absorption, resulting in better broiler growth that supports the current experiment. This finding is similar to those of other researchers [26] who have studied the fermentation of feed using *S. cerevisiae* and feeding broilers.

**Table 5.** Analysis of cost/kg live weight (US$) of broiler receiving different dietary group.

Parameters	Groups					
	**DF**	**MF**	**Y-DF**	**Y-MF**	**YF-MF**	**Sig.**
Feed cost (USD/kg LW)	0.48^c^ ± 0.94	0.43^d^ ± 0.2	0.55^b^ ± 1.6	0.54^b^ ± 0.9	0.64^a^ ± 1.3	**
*Relative to 1*	*100*	*90*	*114*	*113*	*134*	
^1^Production cost (USD/ kg LW)	1.01^b^ ± 0.75	0.96^c^ ± 1.2	1.08^a^ ± 1.9	1.00^b^ ± 1.4	0.96^c^ ± 1.1	**
*Relative to 1*	*100*	*95*	*107*	*99.2*	*95*	
^2^Profit (USD/kg LW)	0.28^b^ ± 0.7	0.33^a^ ± 1.6	0.22^c^ ± 1.9	0.29^b^ ± 1.1	0.34^a^ ± 1.1	**
*Relative to 1*	*100*	*118*	*77*	*102*	*118*	

**= highly significant at 1% level (*p* < 0.01);^ a,b,c,d^ Means bearing uncommon superscripts in a row differ significantly.^1,^ Calculating chick cost (0.46$/bird; Feed cost in T_1_ and T_2_=0.36$/kg. And T_3_, T_4_, T_5_=0.42$/kg).^2,^ assuming sale revenue (1.31 $/kg live weight).DF: Dry Feed; MF: Moist Feed; Y-DF: Yeast added Dry Feed; Y-MF: Yeast added Moist Feed; YF-MF: Yeast Fermented Moist Feed; Values of different variables under different program indicates (Mean ± SD).

Although moist feeding had no major impact on the growth performance and FI of the broiler in the present study, it slightly enhanced these parameters compared to DF. This finding is similar to that of Emadinia et al. [27], who observed that MF has no significant effect on the growth performance of the broiler. However, according to other researchers, wet feed for broilers can increase FI during the day in a hot climate [28] and also improve body weight gain and FCR because adding water to the diet before intake helps digest the feed immediately after feeding and increases the passing rate as well as digestion of feed [29]. However, in the current study, the growth performance and FI were non-significant, which may be due to the effect of the winter season during the feeding trial, which was conducted from November to December 2021.

When yeast (*S. cerevisiae*) is added, the stomach empties more quickly, allowing for a greater intake of food [30] and also helping to balance the microbiota of the gastrointestinal tract, which is crucial for the early growth of the gut and results in greater FI in broilers [28]. Better FCR after feeding a fermented and non-fermented moist diet (Table 3) might be due to the inclusion of *S. cerevisiae* may have improved ileal digestibility and maintained normal microbiota.

Carcass characteristics such as liver, kidney, and shank were significantly increased on the yeast (*S. cerevisiae*) fermented moist diet (YF-MF) compared to other diets (Table 4). The application of *S. cerevisiae* as a nutritional agent may enhance the digestion and absorption of other nutrients like vitamins and minerals that may be correlated with the increase in carcass traits and reduce the negative effect of toxins on carcass characteristics and organ weights in broiler chickens [31].

Yeast (*S. cerevisiae*) fermented wet feed significantly improved bone mineralization in broilers (Fig. 2) because yeast acts as a possible carrier of the phytase enzyme, so yeast-fermented feed improves phosphorus availability to birds [32]. MOS of the yeast cell wall obtain a positive response to Ca absorption and retention [10], whereas Han et al. [33] reported that fermented products contain oligosaccharides that increase the tibia Ca content of broilers. Other researchers, including Swiatkiewic et al. [34] and Kidd et al. [35], also stated a similar statement: tibia ash percentage increased with the supplementation of yeast (*S. cerevisiae),* which helps reduce phytate phosphorus, and fermented feed increased bone mineralization in broilers.

*Saccharomyces cerevisiae* fermented moist feed reduced the production cost of the broiler since the profit margin was highest after feeding fermented wet feed compared to other groups. Feeding YF-MF is a current, cost-effective method to improve the nutritional quality of feed that ultimately increases growth performance. The limitation of the study is using a mash diet, which is not followed by commercial farmers. So, further study should be conducted for commercial scale fermentation, may be fermentation of commercial diet before feeding at farm level.

## Conclusion

Fermentation of complete feed using yeast (*S. cerevisiae*) causes favorable chemical changes of feed, which also improves the nutritive value of feed and is found beneficial for feeding broilers since it improves weight gain and feed efficiency as well as increased tibia ash content and carcass weight. It also becomes cost-effective to use in a broiler diet.

## List of Abbreviations

*S. cerevisiae, Saccharomyces cerevisiae;* %, Percentage; ºC, Degree Centigrade; $, US Dollar.
